# Whole genome sequencing of *Shigella sonnei* through PulseNet Latin America and Caribbean: advancing global surveillance of foodborne illnesses

**DOI:** 10.1016/j.cmi.2017.03.021

**Published:** 2017-11

**Authors:** K.S. Baker, J. Campos, M. Pichel, A. Della Gaspera, F. Duarte-Martínez, E. Campos-Chacón, H.M. Bolaños-Acuña, C. Guzmán-Verri, A.E. Mather, S. Diaz Velasco, M.L. Zamudio Rojas, J.L. Forbester, T.R. Connor, K.H. Keddy, A.M. Smith, E.A. López de Delgado, G. Angiolillo, N. Cuaical, J. Fernández, C. Aguayo, M. Morales Aguilar, C. Valenzuela, A.J. Morales Medrano, A. Sirok, N. Weiler Gustafson, P.L. Diaz Guevara, L.A. Montaño, E. Perez, N.R. Thomson

**Affiliations:** 1) University of Liverpool, Department of Functional and Comparative Genomics, Liverpool, England, United Kingdom; 2) Wellcome Trust Sanger Institute, Pathogen Variation Programme, Hinxton, England, United Kingdom; 3) Instituto Nacional de Enfermedades Infecciosas, ANLIS, Buenos Aires, Argentina; 4) Instituto Costarricense de Investigación y Enseñanza en Nutrición y Salud (Inciensa), Costa Rica; 5) Programa de Investigación en Enfermedades Tropicales, Escuela de Medicina Veterinaria, Universidad Nacional, Heredia, Costa Rica; 6) Centro de Investigación en Enfermedades Tropicales, Facultad de Microbiología, Universidad de Costa Rica, San José, Costa Rica; 7) University of Cambridge, Department of Veterinary Medicine, Cambridge, England, United Kingdom; 8) National Institute of Heath, Lima, Peru; 9) Organisms and Environment Division, Cardiff University School of Biosciences, Sir Martin Evans Building, Cardiff, Wales, United Kingdom; 10) Centre for Enteric Diseases, National Institute for Communicable Diseases and Faculty of Health Sciences, University of the Witwatersrand, Johannesburg, South Africa; 11) Department of Bacteriology, National Institute of Hygiene ‘Rafael Rangel’, Ciudad University, Los Chaguaramos, Venezuela; 12) Molecular Genetics Laboratory, Institute of Public Health of Chile, Santiago, Chile; 13) Department of Foodborne Diseases, National Health Laboratory of Guatemala, Laboratorio Nacional de Salud, Barcenas, Guatemala; 14) Bacteriology Laboratory, Departamento de Laboratorios de Salud Pública (DLSP), Ministerio de Salud Pública (MSP), Montevideo, Uruguay; 15) Department of Bacteriology, Laboratorio Central de Salud Pública, Asuncion, Paraguay; 16) Grupo de Microbiología, Instituto Nacional de Salud, Bogotá, Colombia; 17) Pan American Health Organization/World Health Organization, Department of Health Emergencies, Washington, DC, United States; 18) London School of Hygiene and Tropical Medicine, London, England, United Kingdom

**Keywords:** Shigellosis, Antimicrobial resistance, Diarrhoeal disease, Epidemiology, South America, Central America, Genomics

## Abstract

**Objectives:**

*Shigella sonnei* is a globally important diarrhoeal pathogen tracked through the surveillance network PulseNet Latin America and Caribbean (PNLA&C), which participates in PulseNet International. PNLA&C laboratories use common molecular techniques to track pathogens causing foodborne illness. We aimed to demonstrate the possibility and advantages of transitioning to whole genome sequencing (WGS) for surveillance within existing networks across a continent where *S. sonnei* is endemic.

**Methods:**

We applied WGS to representative archive isolates of *S. sonnei* (*n* = 323) from laboratories in nine PNLA&C countries to generate a regional phylogenomic reference for *S. sonnei* and put this in the global context. We used this reference to contextualise 16 *S. sonnei* from three Argentinian outbreaks, using locally generated sequence data. Assembled genome sequences were used to predict antimicrobial resistance (AMR) phenotypes and identify AMR determinants.

**Results:**

*S. sonnei* isolates clustered in five Latin American sublineages in the global phylogeny, with many (46%, 149 of 323) belonging to previously undescribed sublineages. Predicted multidrug resistance was common (77%, 249 of 323), and clinically relevant differences in AMR were found among sublineages. The regional overview showed that Argentinian outbreak isolates belonged to distinct sublineages and had different epidemiologic origins.

**Conclusions:**

Latin America contains novel genetic diversity of *S. sonnei* that is relevant on a global scale and commonly exhibits multidrug resistance. Retrospective passive surveillance with WGS has utility for informing treatment, identifying regionally epidemic sublineages and providing a framework for interpretation of prospective, locally sequenced outbreaks.

## Graphical abstract

**Figure:**
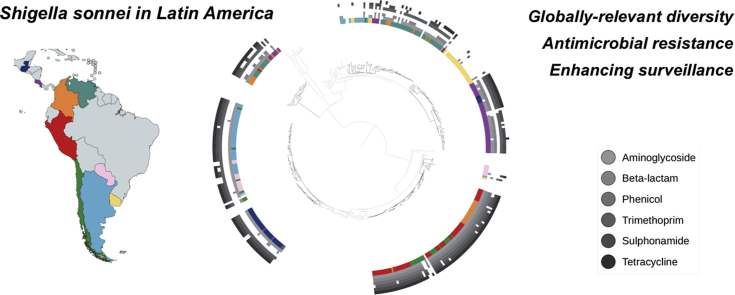

## Introduction

*Shigella* are globally important bacteria, causing more than 190 million diarrhoeal disease cases and 65 796 deaths annually, 18 million and 1023 of which, respectively, occur in the Americas [1], [2]. In Latin America (LA), *S. sonnei* is a common cause of diarrhoeal disease (mainly in children [3], [4], [5]) and is variably resistant to commonly used antimicrobials [6], [7]. Explosive outbreaks still occur (e.g. a 900-case epidemic of *S. sonnei* in Argentina in 2016; http://dx.doi.org/10.1101/049940); and increases in endemic *S. sonnei* prevalence are also reported (http://www.binasss.sa.cr/diarreas2014.pdf), mirroring trends in other economically developing areas [8]. In addition to local transmission, new phylogenetic lineages of *S. sonnei* can disseminate nationally and spread internationally within two to three decades [9], [10]. Given its worldwide distribution, increasing importance and international transmission, it is unsurprising that *S. sonnei* is under surveillance through PulseNet International [11].

PulseNet Latin America and Caribbean (PNLA&C) is a regional network that contributes to PulseNet International, a public health network of >120 laboratories in >80 countries that has performed surveillance of foodborne illnesses for 20 years [11]. PNLA&C laboratories use common molecular subtyping techniques and share their results and associated epidemiologic information through a regional database to facilitate early identification of disease outbreaks in an increasingly globalized world [12]. Owing to the increased resolution compared to traditional techniques (e.g. pulsed-field gel electrophoresis, PFGE), PulseNet International is currently transitioning to the use of whole genome sequencing (WGS) [13].

WGS has been applied to subtype *S. sonnei* effectively: a species-defining study identified four main phylogenetic lineages that were further split into sublineages containing isolates of similar geographical origins [14]. Subsequent WGS studies of *S. sonnei* have demonstrated the emergence of sublineages of public health importance at national and international levels, often driven by the acquisition of antimicrobial resistance (AMR) [9], [10], [14], [15]. In a strict public health setting, Public Health England researchers have used WGS to identify epidemiologic clusters of *S. sonnei* because existing subtyping techniques (phage typing) provided poor lineage discrimination [16]. WGS has also been used to predict AMR phenotypes, with high (e.g. >95%) specificities and sensitivities reported for other *Enterobacteriaceae,* including *Escherichia coli, Campylobacter* and *Salmonella* [17], [18], [19]. Collectively, these studies suggest that the application of WGS within international surveillance networks, such as PNLA&C, can enhance outbreak detection and surveillance for *S. sonnei* and its AMR determinants.

The strength of surveillance frameworks lies in both the use of common techniques and large reference databases; hundreds of thousands of PFGE-subtyped pathogen profiles exist within PulseNet International. Populating these databases with WGS data from passive and active surveillance programs will promote the continued success of international surveillance. In this study, members of PNLA&C worked collaboratively towards this goal by generating a WGS overview of *S. sonnei* in the region.

## Materials and Methods

### Clinical isolates

#### LA archive isolates

**Fig. 1:**
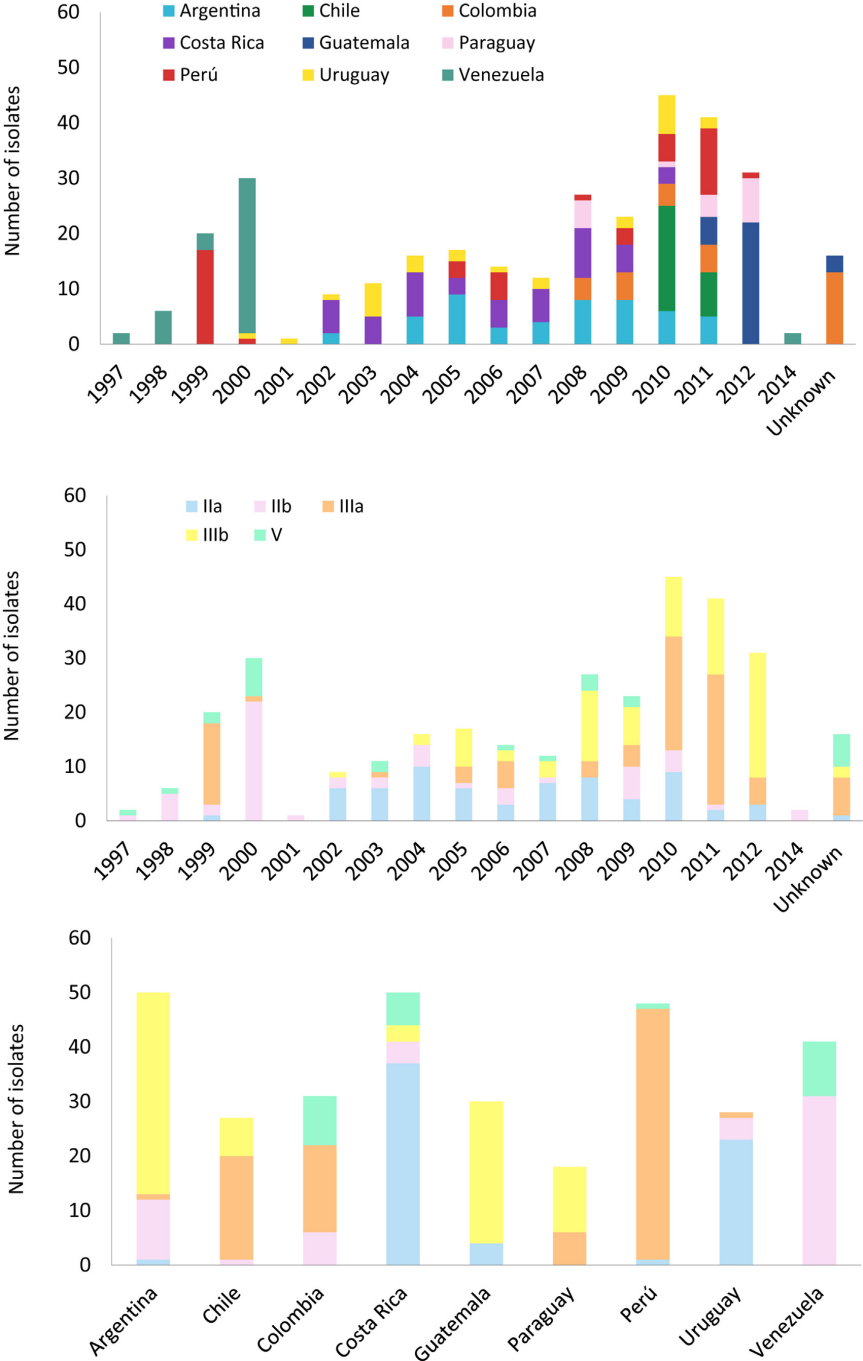
Distribution of 323 Latin American *Shigella sonnei* isolates sequenced as part of this study by year and country (top), sublineage designation and year (middle), and sublineage and country (bottom).

To construct a regional overview of *S. sonnei* across LA, WGS data were generated from 323 archived clinical isolates of *S. sonnei* collected over 19 years from nine countries (Table 1, Fig. 1). Each national PNLA&C partner was responsible for selecting isolates from its own archives with the aim of achieving diversity with respect to the following: PFGE profile, year of isolation, AMR profile, disease manifestation and PFGE profile linkage to outbreaks of disease or sporadic cases. Metadata associated with the isolates frequently included the year of collection, AMR susceptibility testing results and geographical information (e.g. patient residential province or address of submitting laboratory). All metadata and results are shown in Supplementary Table S1.

**Table 1:** *Shigella sonnei* isolates studied

Study	Country	Years	*n*
Latin America	Argentina	2002–2011	50
	Chile	2010–2011	27
	Colombia	2008–2011	31
	Costa Rica	2002–2010	50
	Guatemala	2011–2012	30
	Paraguay	2008–2012	18
	Peru	1999–2012	48
	Uruguay	2000–2011	28
	Venezuela	1997–2014	41
Global	Many	1943–2008	116

	**Total**	**NA**	**439**

#### Global context isolates

To set this regional overview in a global context, publically available sequence data from global reference isolates (*n* = 116) of *S. sonnei* were also included (Supplementary Table S1). These comprise temporally and geographically diverse (samples from four continents collected between 1943 and 2008) isolates used to define the population structure of *S. sonnei* [14].

#### Argentine outbreak isolates

To demonstrate the utility of the LA regional overview for investigating national outbreaks, WGS data generated at the PNLA&C reference laboratory (ANLIS) from three Argentinian outbreaks (*n* = 16 isolates) of *S. sonnei* were also used. Previously reported at a national level (http://dx.doi.org/10.1101/049940), these isolates were from outbreaks in 2010 (*n* = 5), 2011 (*n* = 3) and 2016 (*n* = 8).

### Genome sequencing and bioinformatics analysis

Archive isolates were sequenced, trimmed and quality checked at the Wellcome Trust Sanger Institute according to in-house protocols [20]. Sequencing data were *de novo* assembled using a custom assembly pipeline [21]. All isolates were assembled into >4MB and <650 contiguous sequences (Supplementary Table S1). Sequencing data and assemblies are publically available at the European Nucleotide Archive; accession numbers are listed in Supplementary Table S1. Argentinian outbreak isolates were sequenced at ANLIS (http://dx.doi.org/10.1101/049940).

To construct the regional overview phylogeny, a multiple sequence alignment was created by mapping the sequence data from 439 taxa (archive and global context isolates) to *Shigella sonnei* Ss046 and its five associated plasmids (5055316 bp) using SMALT, followed by removal of repeat regions and mobile elements (7210310 bp) [14] and regions of recombination (7074 sites) [22], resulting in a final alignment of 13 988 variant sites. A maximum likelihood phylogeny with 100 bootstraps was then inferred [23]. Phylogenetic analysis incorporating outbreak isolates was conducted similarly (final alignment 14 075 variant sites).

For analysis of sequences related to AMR, AMR genes were detected on assembled sequences [24] and cross-referenced with phylogeny, contiguous sequence length and traditional comparative genetic approaches including Artemis, BLAST (against National Center for Biotechnology Information (NCBI) reference databases and locally) and the Artemis comparison tool, as previously described [9] to determine the presence of known AMR determinants in shigellae. Single nucleotide polymorphisms (SNPs) in known quinolone-resistance determining regions, including *gyrA* positions 83, 87 and 211, and *parC* positions 80 and 84 [25], were retrieved.

Approximate longitude and latitude of locations were deduced, and phylogeographic analysis was visualized by MicroReact [26]. Figtree and iTOL were used for additional visualizations [27].

## Results

**Fig. 2:**
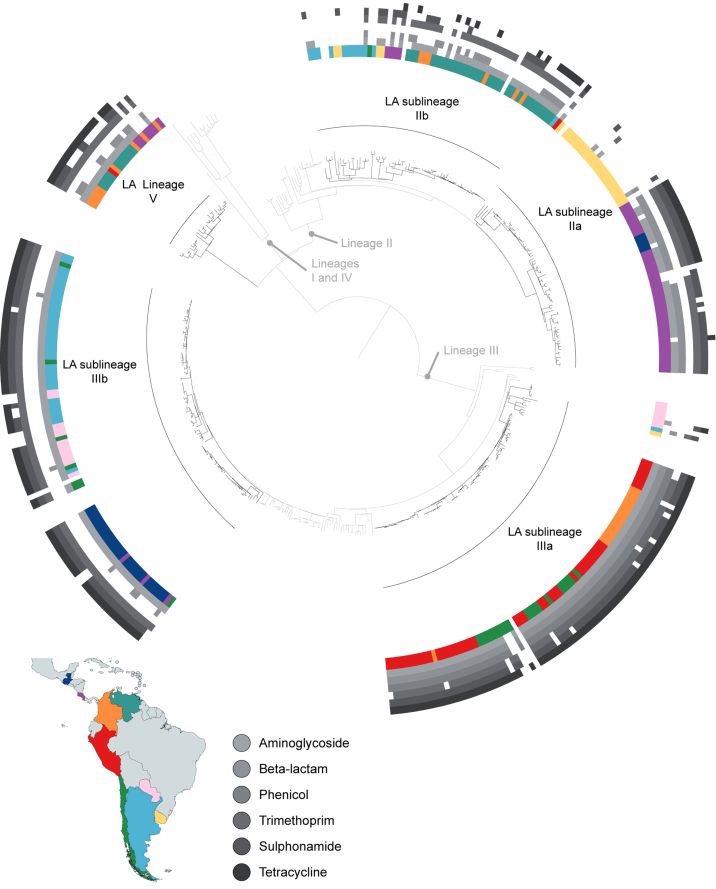
Genomic portrait of Latin American (LA) *Shigella sonnei* in context. Maximum likelihood phylogenetic tree of *S. sonnei* showing 323 LA isolates. LA sublineages are shown in black and labelled with adjacent arcs; topology unlinked with LA isolates is shown in grey, with lineages labelled to the inner of the tree. Country of origin of LA isolates is shown on internal track at tips of tree, coloured according to map. Presence of predicted antimicrobial resistance is shown in outer tracks according to inlaid key. Global reference isolates in tree do not have country or resistance labels.

Phylogenetic analysis of LA *S. sonnei* was conducted to define its population structure within the known four-lineage context of *S. sonnei.* This divided the LA isolates into a new genetic lineage and four genetic sublineages of variable diversity. The new lineage comprised 26 (8%) of the 323 archive isolates and was designated lineage V (Fig. 1, Fig. 2, Table 1). Since its detection in this study, lineage V isolates have been detected in South Africa (Supplementary Fig. S1) and the United Kingdom (Baker et al., in preparation). The remaining archive isolates clustered within lineages II (*n* = 123, 38%) and III (*n* = 174, 54%). The archive isolates in lineage II were further subdivided into the sublineages IIa and IIb (Table 1, Fig. 1, Fig. 2). LAIIa and IIb were phylogenetically distinct from the previously described South America II sublineage (Supplementary Fig. S2). The archive isolates in lineage III were similarly subdivided into sublineages IIIa and IIIb, which were expansions of previously described sublineages (Table 1, Supplementary Fig. S2), with IIIb being part of the multidrug-resistant (MDR) Global III sublineage of *S. sonnei* that expanded globally after the acquisition of AMR [14]. The four LA sublineages had variable genetic diversity with, for example, the maximum multiple sequence alignment pairwise distance between any two isolates in IIIb being 172 SNPs compared to 309 SNPs for IIb (Table 2). *S. sonnei* isolates belonging to lineages I and IV were not found in this study.

**Table 2:** Genomic and epidemiologic features of Latin American *Shigella sonnei*

Characteristic	Lineage	Sublineage			
	V	LAIIa	LAIIb	LAIIIa	LAIIIb
Isolate features					
No. of isolates	26 (8%)	66 (20%)	57 (18%)	89 (28%)	85 (26%)
Years	1997–2009	1999–2012	1997–2014	1999–2012	2002–2012
No. of countries	4	5	6	6	5
Pairwise distances (SNPs)					
Average	134	176	161	95	105
Median	160	208	210	95	118
Largest distance	221	295	309	292	172
Previous sublineage name^a^	—	Unnamed	Unnamed	South America (III)	Africa/South America, within Global III

LA, Latin America; SNP, single nucleotide polymorphism.

a According to [14].

**Fig. 3:**
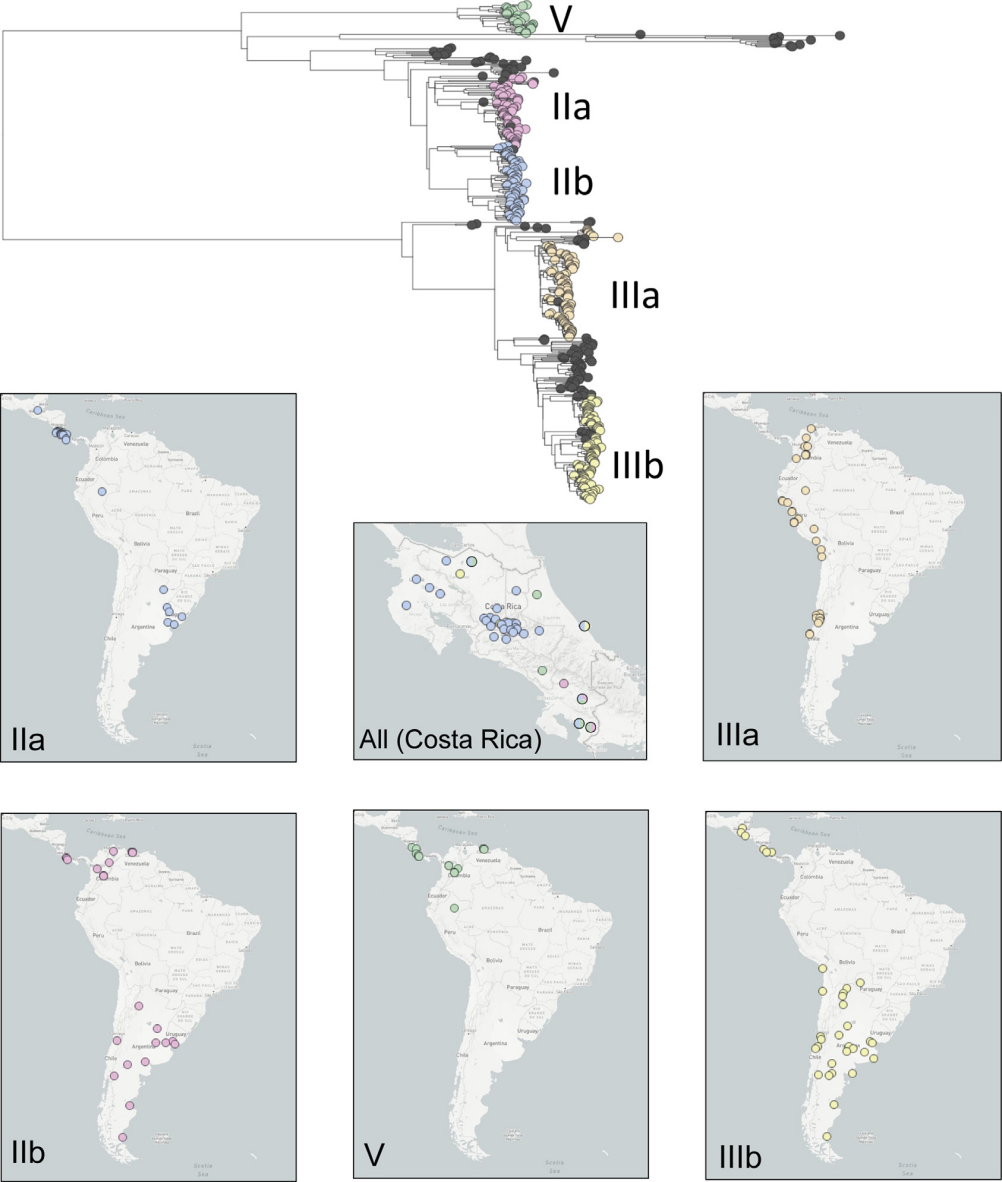
Phylogeography of Latin American (LA) *Shigella sonnei.* Midpoint rooted phylogenetic tree with taxa coloured by LA lineage or sublineage (reference isolates shown in grey). Distribution of each lineage or sublineage across LA is shown in maps, and for all sublineages and lineages in Costa Rica.

The regional phylogenetic overview provides a nomenclature for discussion and interpretation of national and regional surveillance patterns of *S. sonnei* in LA. For that reason, the geospatial information and phylogenetic results of the archived isolates were loaded into a MicroReact project (http://microreact.org/project/Shigella_sonnei_in_Latin_America). This public resource can be used interactively by end users to display and filter the results of this study on the basis of time, phylogeny and geography to highlight, for example, that sublineages were not uniformly distributed around LA or individual countries (Fig. 3).

The isolates within an LA lineage or sublineage were temporally and geographically diverse. Each lineage or sublineage contained isolates collected in multiple years over the course of the study, ranging from 10 years for IIIb to the entire 17 years for IIb (Fig. 1, Table 2). No obvious shifts in the presence of the LA lineage or sublineages were seen over time, apart from IIIb possibly predominating in later years (Fig. 1). The LA lineage and sublineages were also diverse with respect to their countries of origin, with each comprising isolates from between four and six countries (Fig. 1, Fig. 2, Table 1). Within a given LA lineage or sublineage, isolates from different countries were frequently intermingled rather than being phylogenetically separated on the basis of geography (often with good phylogenetic support; Supplementary Fig. S5), indicating that international transmission across the region may be occurring.

**Fig. 4:**
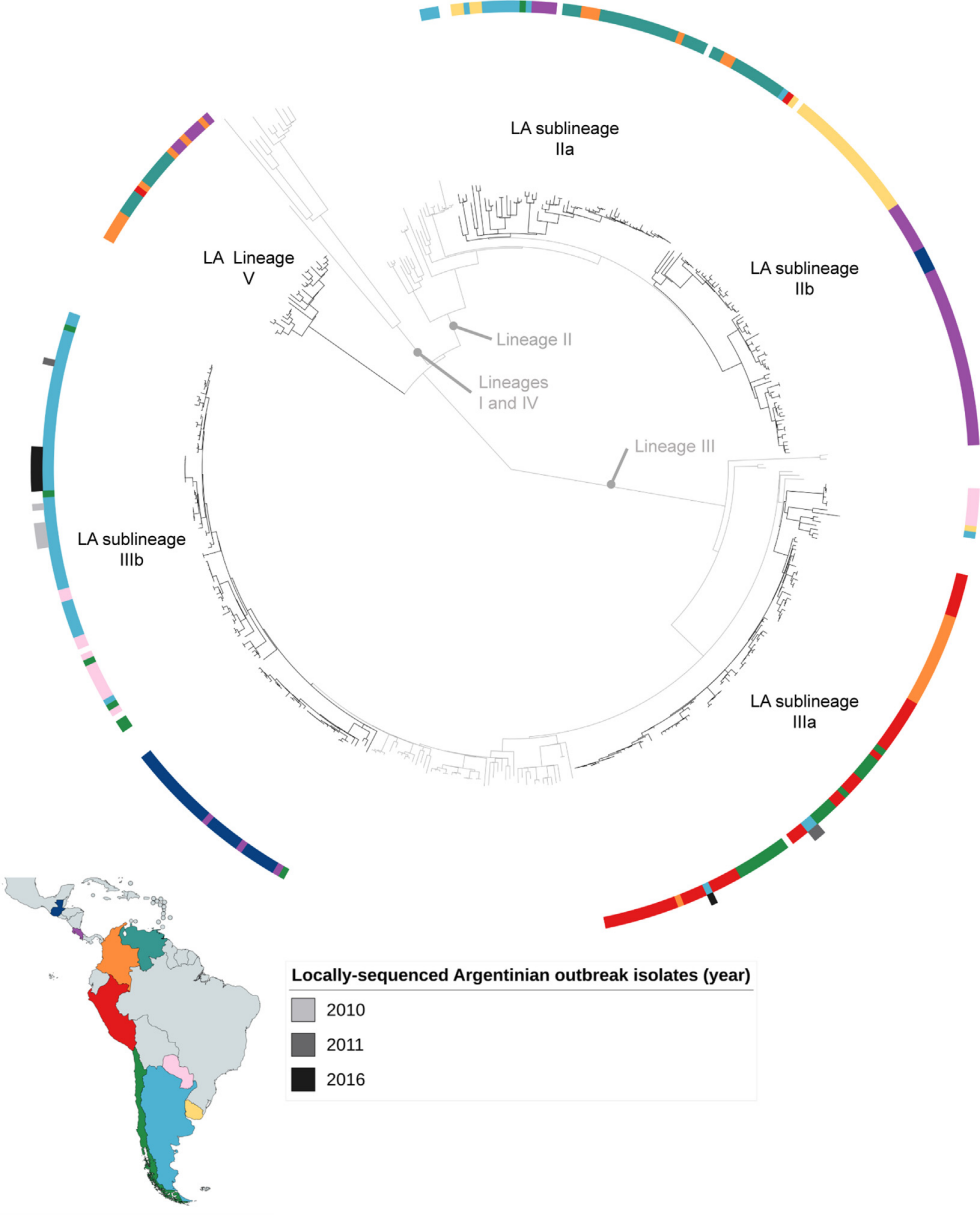
Argentine *Shigella sonnei* outbreaks in context of Latin American regional overview. Midpoint rooted phylogenetic tree shows phylogenetic positions of outbreak isolates from outbreaks in 2010, 2011 and 2016 using data generated locally in Argentina. Tree is labelled similarly to Fig. 2, with additional outbreak isolates being coloured by year (according to inlaid key) in outermost track exterior to country labels (coloured according to map).

To demonstrate the utility of this data for contextualising new outbreaks, we performed further phylogenetic analysis with additional isolates from *S. sonnei* outbreaks in Argentina. This confirmed that the Argentinian outbreaks in 2010 and 2011 were caused by phylogenetically distinct *S. sonnei* with distinct AMR profiles (http://dx.doi.org/10.1101/049940). Here this is demonstrated by the majority of isolates from the 2011 outbreak belong to sublineage LAIIIa and those from 2010 and 2016 belonging to sublineage LAIIIb (Fig. 4). Notably, however, the 2011 and 2016 isolates fell into multiple sublineages, indicating that the outbreaks may have multiple epidemiologic origins.

Predicted AMR phenotypes correlated well with AMR testing data (94.3% sensitivity, 99.4% specificity of 330 available phenotypes; Supplementary Table S1); these are the results presented throughout this text. MDR (resistance to three or more antimicrobial classes) was common among the isolates (77%, 249 of 323), with isolates being resistant to between 0 and 7 antimicrobial agents (mean 3.7, mode and median 4) (Table 3, Supplementary Table S1, Fig. 2). No relationship of increasing AMR over time was detected (Supplementary Fig. S4). Macrolide resistance and an extended-spectrum β-lactamase gene were found in individual isolates, conferred by the azithromycin resistance gene *mphA* in a IIIb isolate, and a lineage V isolate containing a *bla*_SHV_*129* gene that conferred resistance to ceftazidime (Supplementary Table S1). Resistance to quinolones was similarly infrequent, being present in only 3% (10 of 323) of isolates (conferred by *gyrA* mutations (*n* = 8) or *qnr* genes (*n* = 2), Supplementary Table S1, Table 3). Resistance to other classes of antimicrobials was more common, with 65 to 82% of isolates being resistant to aminoglycosides (streptomycin), trimethoprim, sulphonamide and tetracycline classes of antimicrobials, and resistance to phenicol and β-lactam classes also being frequently detected (25 and 48% of isolates respectively) (Fig. 2, Table 3). These resistances were encoded by a variety of AMR genes (Supplementary Table S1, Supplementary Fig. S3).

**Table 3:** Predicted resistance phenotypes and major resistance determinants

Characteristic	Lineage	Sublineage				All (*n* = 323)
	V (*n* = 26)	LAIIa (*n* = 66)	LAIIb (*n* = 57)	LAIIIa (*n* = 89)	LAIIIb (*n* = 85)	
Predicted resistant						
Aminoglycoside	81%	58%	70%	90%	100%	82%
β-Lactam	42%	59%	35%	90%	7%	48%
Phenicol	0	2%	0	89%	0	25%
Trimethoprim	85%	61%	68%	81%	100%	80%
Sulphonamide	46%	62%	30%	83%	87%	67%
Tetracycline	73%	30%	25%	90%	91%	65%
Macrolide	0	0	0	0	1%	0
Quinolone	0	0	0	9%	0	3%
ESBL	4%	0	0	0	0	0
MDR						
MDR	81%	62%	51%	91%	91%	77%
Average no. of AMR phenotypes per isolate	3.3	2.7	2.3	5.3	3.9	3.7
No. of unique resistance profiles	9	11	13	11	6	30
Previously described major AMR determinants						
Aminoglycoside	—	Int2/Tn7 with *bla*	Int2/Tn7	SRL	Int2/Tn7 and SpA	—
β-Lactam	—	Int2/Tn7 with *bla*	—	SRL	—	—
Phenicol	—	—	—	SRL	—	—
Trimethoprim	—	Int2/Tn7	Int2/Tn7	SRL	SpA	—
Sulphonamide		—	—	pABC-3	Int2/Tn7	—
Tetracycline		—	—	pABC-3	SpA	—
Macrolide		—	—	—	—	—
Quinolone		—	—	*gyrA* SNPs, qnrS genes	—	—

AMR, antimicrobial resistance; ESBL, extended-spectrum β-lactamase; LA, Latin America; MDR, multidrug resistant; SNP, single nucleotide polymorphism.

Notably, the distribution of resistance against an antimicrobial class was not uniform among the sublineages (Fig. 2). For example, predicted β-lactamase resistance in sublineage IIIa was 90% compared to just 7% for sublineage IIIb (p <0.01), and while IIIa and IIIb had ∼90% resistance to tetracycline, sublineages IIb and IIa had between 25 and 30% tetracycline resistance (Table 3). The presence of resistance towards a variety of antimicrobials gave rise to an AMR profile (i.e. antibiogram) in each isolate (Fig. 2), with each sublineage containing isolates of between six and 13 different AMR profiles. However, in the case of sublineages IIIa and IIIb, single AMR profiles dominated, with one profile being present among 69 and 79% (respectively) of the isolates in the sublineage, and other AMR profiles being present in less than 9% of isolates in the sublineage (Supplementary Table S1, Fig. 2). The dominant AMR profiles in each of IIIa and IIIb were determined by the presence of chromosomal and plasmid-encoded AMR genes conferring resistance to multiple antimicrobial classes (Table 3). Specifically, sublineage IIIb carried the chromosomal Int2/Tn7 resistance determinant and the SpA plasmid, and sublineage IIIa carried the chromosomal *Shigella* resistance locus (SRL) and a variant plasmid of SpA, pABC-3, on which the aminoglycoside resistance gene *strA* has been interrupted by the acquisition of a trimethoprim resistance-conferring gene, *dfrA14* [28]. In contrast to the presence of a dominant AMR profile in sublineages IIIa and IIIb, sublineages IIa, IIb and lineage V contained at least three AMR profiles that were present in ≥15% of the isolates (Supplementary Table S1).

## Discussion

Here we have created a resource of novel WGS diversity of *S. sonnei* from LA relevant to public health surveillance on regional and global scales. We report the identification of a new global lineage (lineage V) [14]; previously undescribed sublineages of lineage II (LAIIa and IIb); and expansions in lineage III, including LAIIIa (previously South America (III)) and IIIb within the MDR Global III lineage. The subsequent detection of lineage V in Europe and Africa testifies to the relevance of this diversity for surveillance on a global scale, and the regional importance of this data is demonstrated here by contextualisation of Argentinian *S. sonnei* outbreaks.

In a previous study, outbreak isolates from Argentina were discriminated at a national level into three WGS sublineages (http://dx.doi.org/10.1101/049940). Building these isolates into this regional overview, the 2010 and 2016 outbreak isolates were contained entirely within the diversity of archive isolates from Argentina, indicating these epidemics were likely from previously circulating strains (Fig. 4). In contrast, the 2011 outbreak isolates were more closely related to IIIa isolates from Peru and Chile than the single IIIa isolate from Argentina, indicating the epidemic may have been subsequent to an importation event. Thus, by providing interpretative context, our results enhance national, regional and global surveillance of *S. sonnei* through publically available sequencing data and MicroReact.

The WGS data were also used to examine AMR in *S. sonnei* across LA. Quinolone and macrolide resistance and genes encoding extended-spectrum β-lactamases were not widespread; they were present in only a handful of isolates. Notably, however, quinolone-resistant isolates in lineage V and sublineage IIIa were outside of the Central Asia III lineage, thought to act as the global reservoir for ciprofloxacin-resistant *S. sonnei* [15]. Resistance against many other classes of antimicrobials (including aminoglycosides, β-lactams, trimethoprim-sulphonamides, phenicol and tetracyclines), however, was common, as was MDR. Common MDR across the sublineages is part of the problem of increasing AMR in *Shigella* and has already lead to the emergence of epidemiologically dominant sublineages elsewhere [9], [10].

This study provides applications to future surveillance as well as retrospective insight on *S. sonnei* epidemiology across LA. The presence of closely related isolates from different countries within an individual sublineage indicates that international transmission of *S. sonnei* occurs across LA, as suggested for the Argentina 2011 outbreak. Also, although not a representative epidemiologic cross section, this study suggests that sublineages IIIa and IIIb are epidemically expanding across LA, driven by AMR. This is supported by the temporal incidence of IIIa and IIIb isolates being weighted towards the latter years of the study and the low phylogenetic diversity in sublineages IIIa and IIIb compared to sublineages IIa and IIb, and lineage V, likely resulting from rapid clonal expansion. Furthermore, the presence of a dominant AMR profile in each lineage is associated with combinations of specific AMR determinants already associated with globally epidemic *S. sonnei.* Specifically, sublineage IIIb falls within the Global III sublineage and contains the Int2/Tn7 resistance determinant and the pSpA resistance plasmid associated with the global expansion of Global III [14]. Similarly, sublineage IIIa contains the chromosomally integrated SRL and pABC-3, two of the major resistance determinants reported in a recent sublineage of *Shigella flexneri 3a* which transmitted globally among men who have sex with men from a possible LA origin [29]. Notably, pABC-3 has been reported in epidemic *S. sonnei* in Chile [28], but the SRL, known as an important AMR determinant in *S. flexneri* and *Shigella dysenteriae* [30], [31], has not been commonly reported in *S. sonnei* and thus represents a worrying addition to the armaments of this pathogen.

There were several limitations to this study. It was retrospective in nature, and we selected intentionally diverse isolates without a case definition or representation relative to the disease burden of each country. Thus, the final overview only approximates proportional representation of the phylogenetic variation and AMR profiles of *S. sonnei* in LA and should be used primarily as a contextual tool for future surveillance. Additionally, using WGS for *Shigella* surveillance relies on isolate culture, which is diagnostically less sensitive than alternative molecular techniques such as quantitative real-time PCR [32]. However, as evidenced here, isolate cultures have a value-added role for epidemiologic, AMR and evolutionary surveillance given the increased insight that can be gained through WGS.

As we exploit novel subtyping techniques for understanding the spread of pathogens, contextual databases need to be rebuilt through regional cooperation and investment in appropriate technologies, facilities and training. This study demonstrates that this is possible within existing infrastructure and surveillance networks, such as PNLA&C. Through collaborative efforts, we created a context for *S. sonnei* across LA showing international transmission and epidemiologically expanding sublineages within this region. We have also shown how this information can be used to place recent outbreaks and increasing levels of AMR into national, regional and global contexts. This information is essential if we are to maintain current surveillance activities and halt the increase and spread of AMR in important bacterial pathogens such as *S. sonnei.*
