# Supplementary figures and images for: Whole genome sequencing of Shigella sonnei through PulseNet Latin America and Caribbean: advancing global surveillance of foodborne illnesses
Source: Clin Microbiol Infect. 2017 Nov;23(11):845–53. doi: 10.1016/j.cmi.2017.03.021 (PMC5667938; doi:10.1016/j.cmi.2017.03.021)

## Slide 1
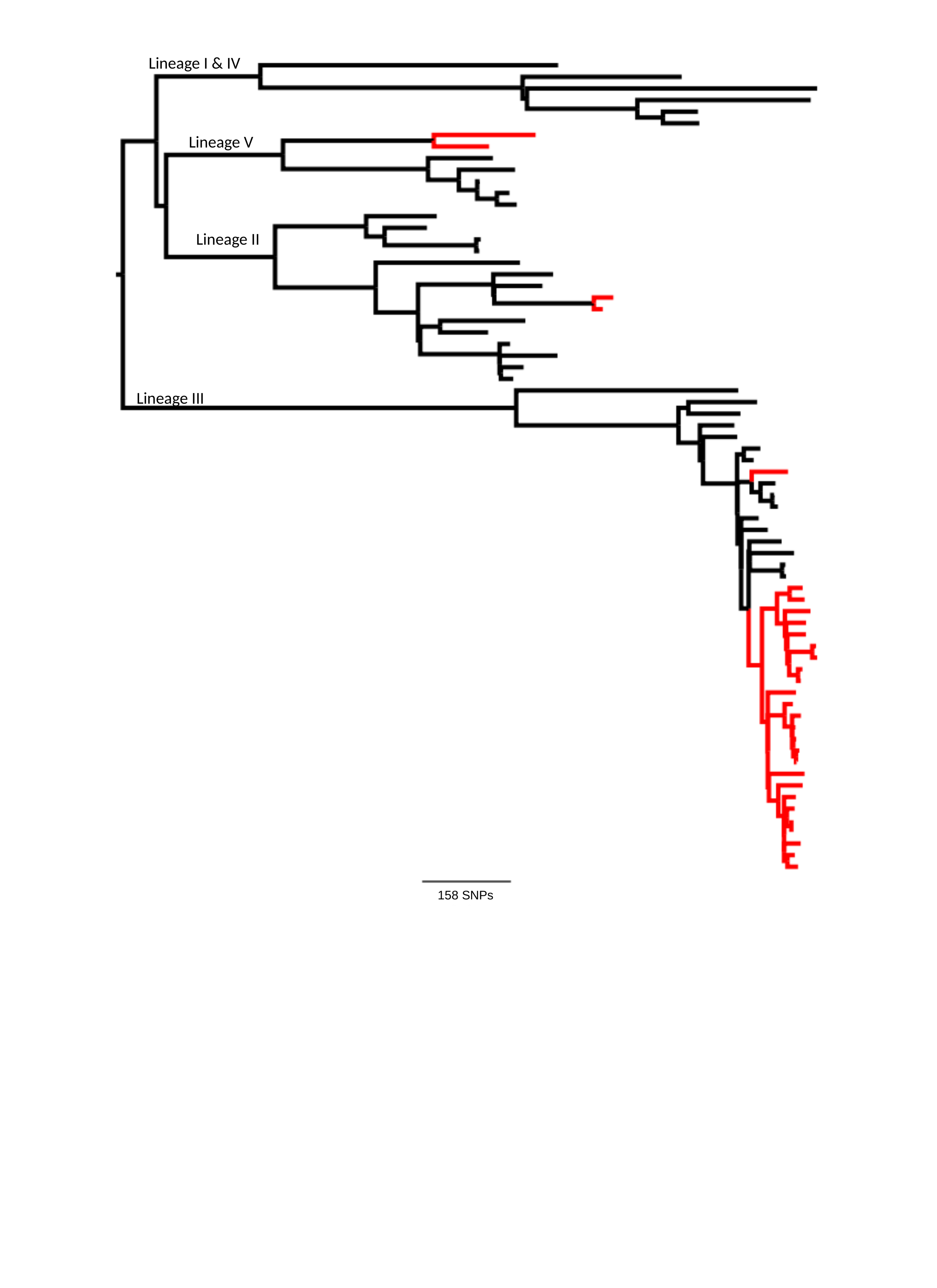

Lineage I & IV
Lineage V
Lineage II
Lineage III
158 SNPs

Supplement: Supplementary file 1 [file mmc1.pptx]

## Slide 1
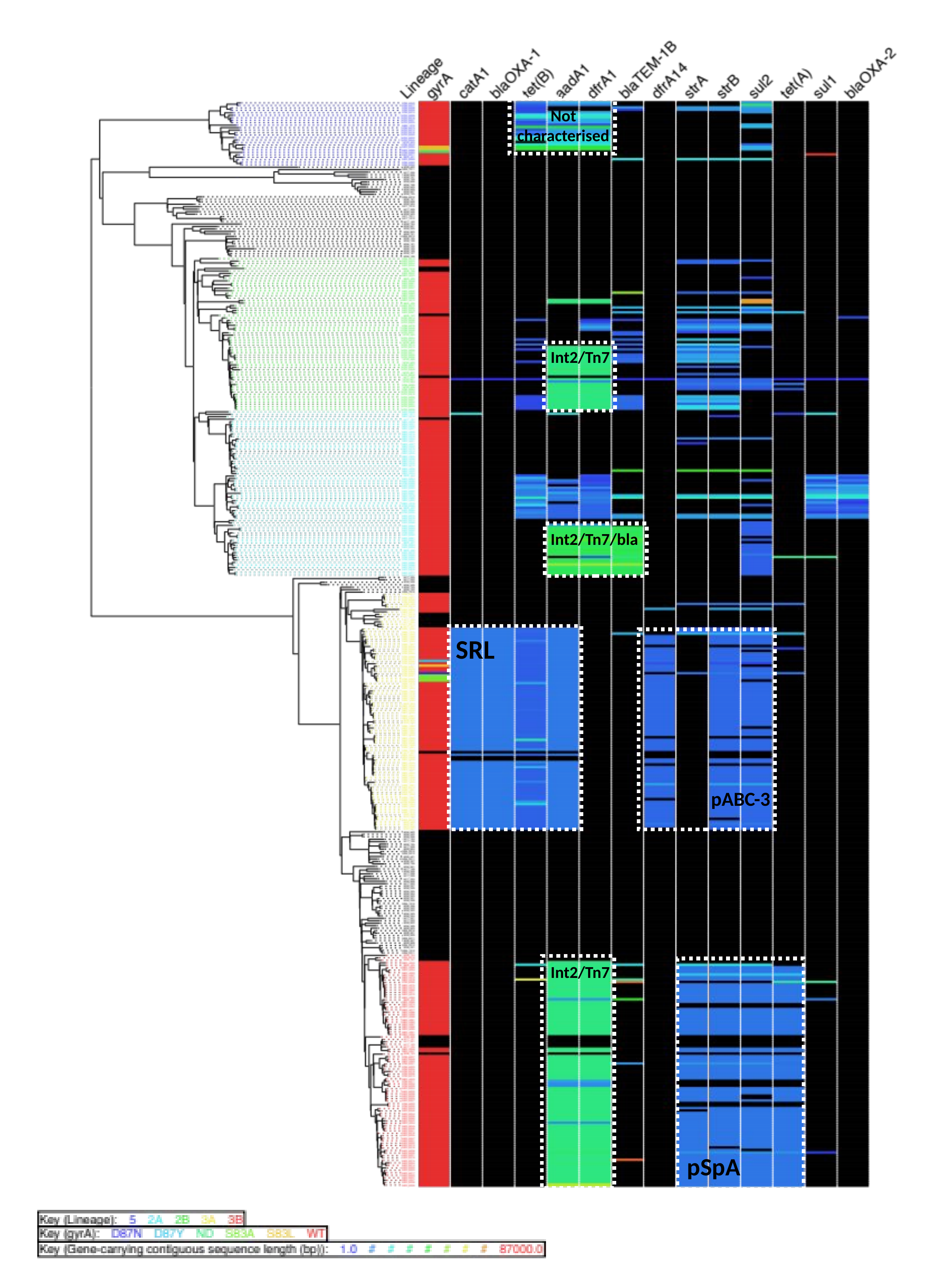

Not characterised
Int2/Tn7
Int2/Tn7/bla
SRL
pABC-3
Int2/Tn7
pSpA

Supplement: Supplementary file 3 [file mmc3.pptx]

IIb

IIa

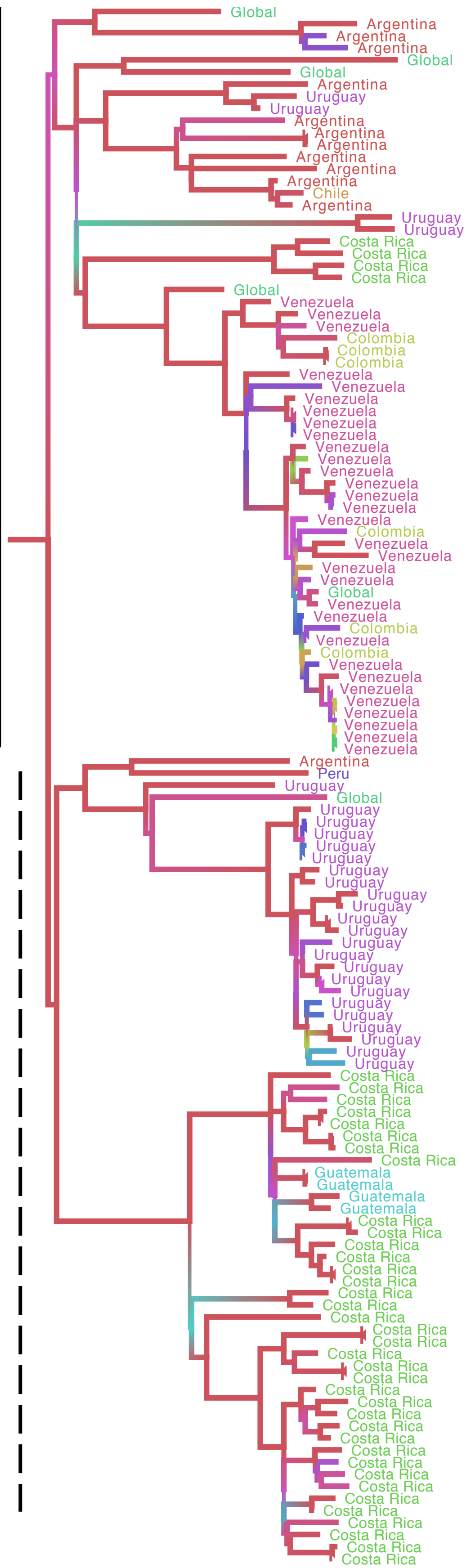

Bootstrap value

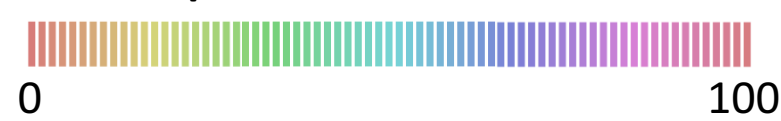

IIIa

IIIb

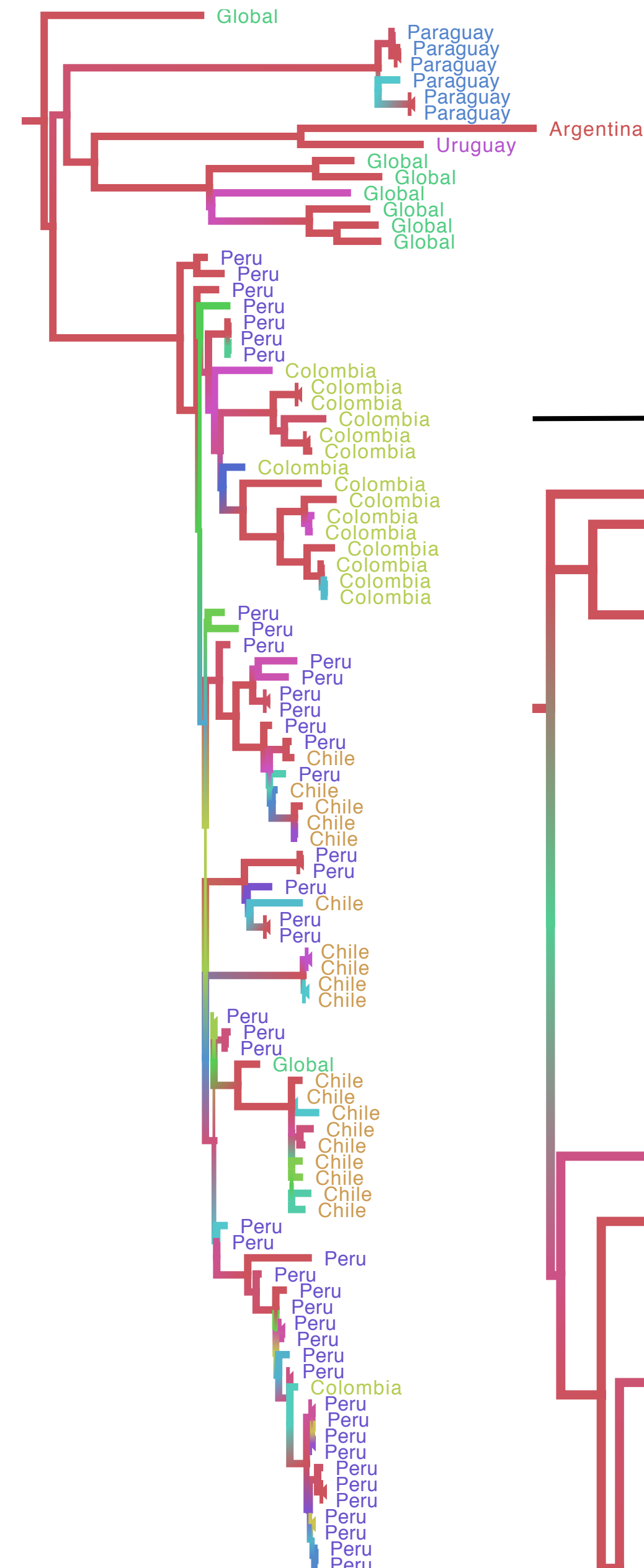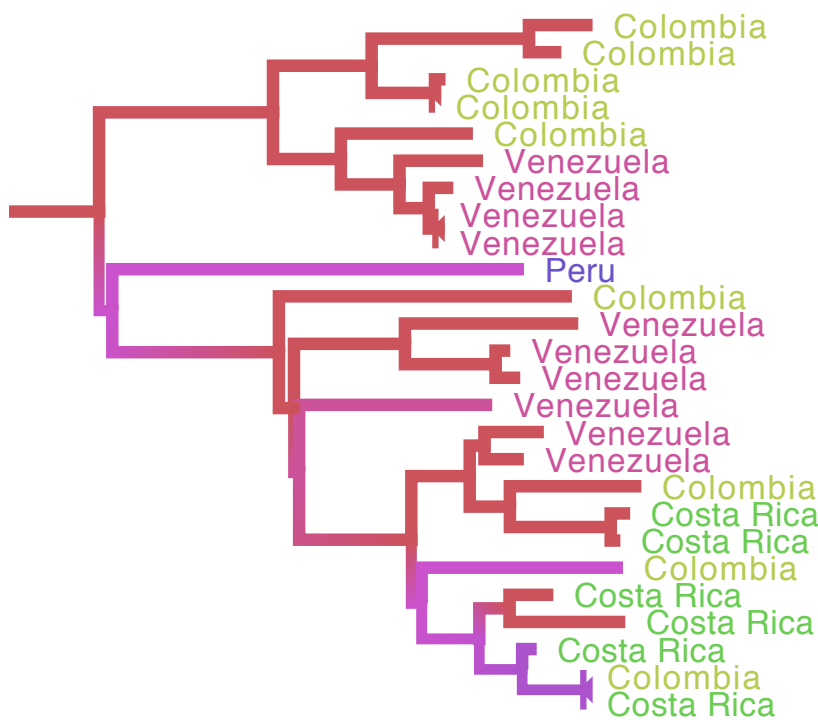

V

Supplement: Supplementary file 5 [file mmc5.pdf]
