# Supplementary material for: Whole genome sequencing of Shigella sonnei through PulseNet Latin America and Caribbean: advancing global surveillance of foodborne illnesses
Source: Clin Microbiol Infect. 2017 Nov;23(11):845–53. doi: 10.1016/j.cmi.2017.03.021 (PMC5667938; doi:10.1016/j.cmi.2017.03.021)
Supplement: Supplementary file 2 [file mmc2.pptx]

## Slide 1
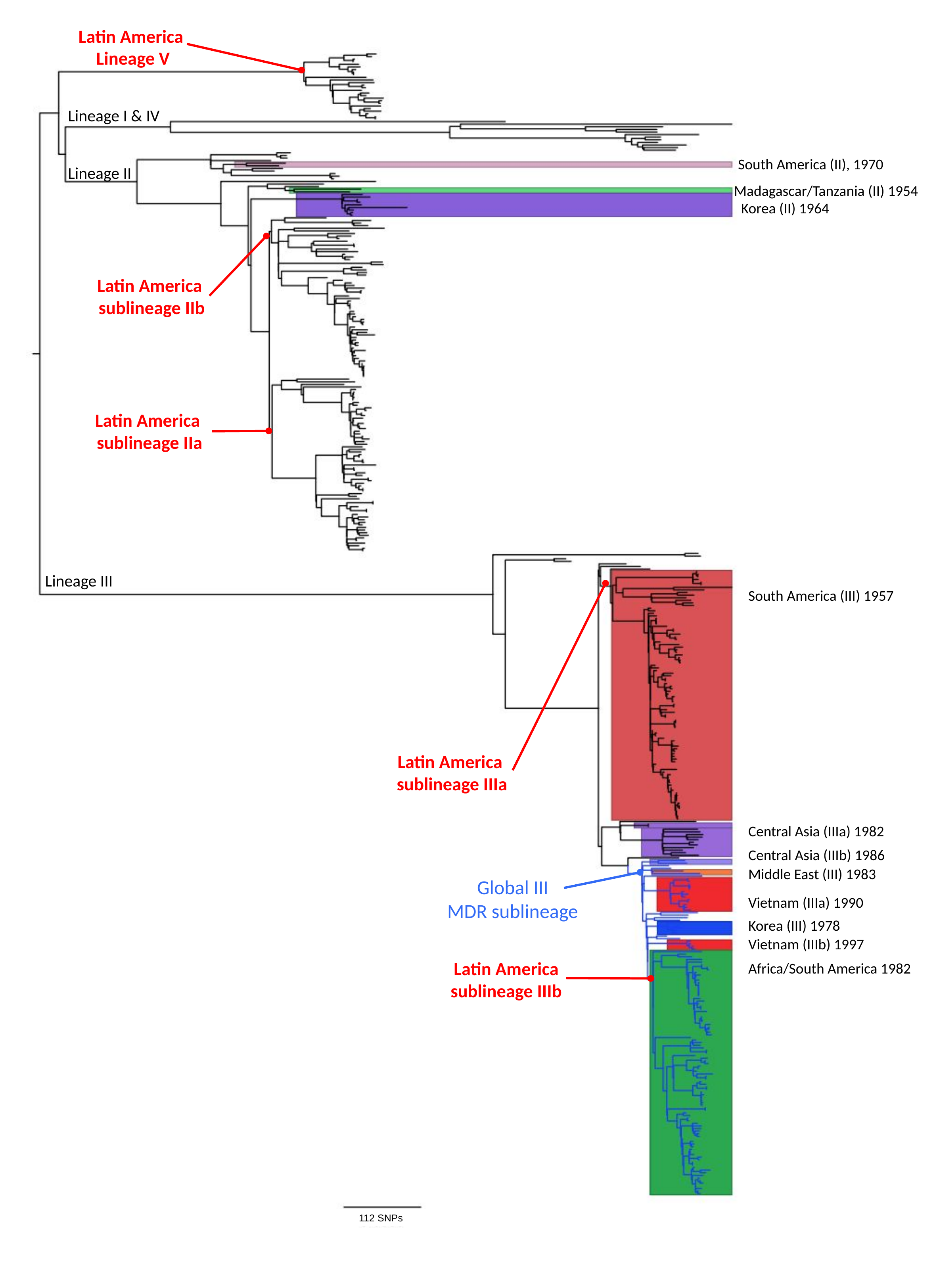

Latin America
Lineage V
Lineage I & IV
South America (II), 1970
Lineage II
Madagascar/Tanzania (II) 1954
Korea (II) 1964
Latin America
sublineage IIb
Latin America
sublineage IIa
Lineage III
South America (III) 1957
Latin America
sublineage IIIa
Central Asia (IIIa) 1982
Central Asia (IIIb) 1986
Middle East (III) 1983
Global III
MDR sublineage
Vietnam (IIIa) 1990
Korea (III) 1978
Vietnam (IIIb) 1997
Latin America
sublineage IIIb
Africa/South America 1982
112 SNPs
