# Supplementary material for: Whole genome sequencing of Shigella sonnei through PulseNet Latin America and Caribbean: advancing global surveillance of foodborne illnesses
Source: Clin Microbiol Infect. 2017 Nov;23(11):845–53. doi: 10.1016/j.cmi.2017.03.021 (PMC5667938; doi:10.1016/j.cmi.2017.03.021)
Supplement: Supplementary file 4 [file mmc4.pptx]

## Slide 1
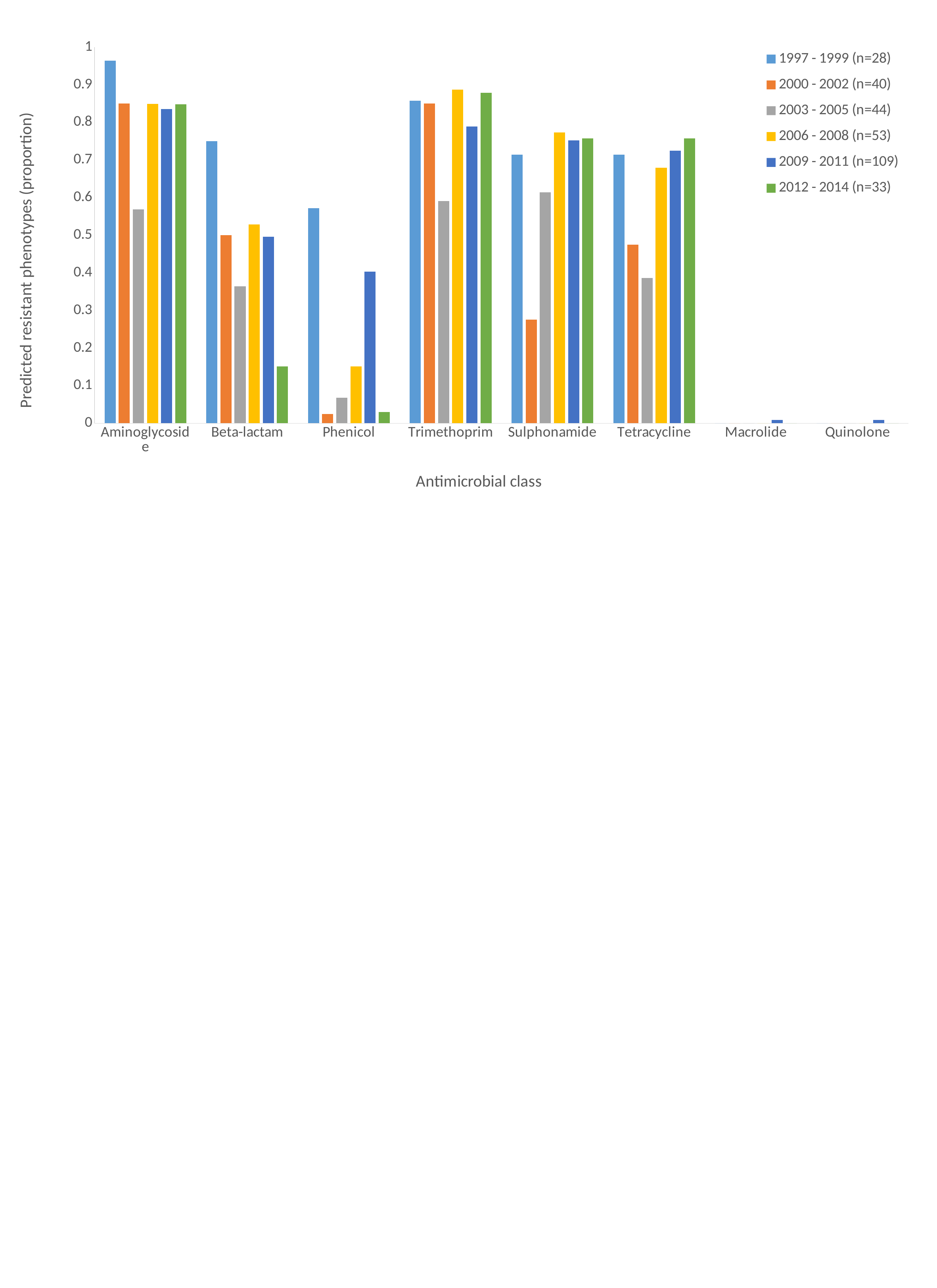

### Chart
| Category | 1997 - 1999 (n=28) | 2000 - 2002 (n=40) | 2003 - 2005 (n=44) | 2006 - 2008 (n=53) | 2009 - 2011 (n=109) | 2012 - 2014 (n=33) |
|---|---|---|---|---|---|---|
| Aminoglycoside | 0.964285714285714 | 0.85 | 0.568181818181818 | 0.849056603773585 | 0.834862385321101 | 0.848484848484848 |
| Beta-lactam | 0.75 | 0.5 | 0.363636363636364 | 0.528301886792453 | 0.495412844036697 | 0.151515151515152 |
| Phenicol | 0.571428571428571 | 0.025 | 0.0681818181818182 | 0.150943396226415 | 0.403669724770642 | 0.0303030303030303 |
| Trimethoprim | 0.857142857142857 | 0.85 | 0.590909090909091 | 0.886792452830189 | 0.788990825688073 | 0.878787878787879 |
| Sulphonamide | 0.714285714285714 | 0.275 | 0.613636363636364 | 0.773584905660377 | 0.752293577981651 | 0.757575757575757 |
| Tetracycline | 0.714285714285714 | 0.475 | 0.386363636363636 | 0.679245283018868 | 0.724770642201835 | 0.757575757575757 |
| Macrolide | 0.0 | 0.0 | 0.0 | 0.0 | 0.0091743119266055 | 0.0 |
| Quinolone | 0.0 | 0.0 | 0.0 | 0.0 | 0.0091743119266055 | 0.0 |
